# Using machine learning for the personalised prediction of revision endoscopic sinus surgery

**DOI:** 10.1371/journal.pone.0267146

**Published:** 2022-04-29

**Authors:** Mikko Nuutinen, Jari Haukka, Paula Virkkula, Paulus Torkki, Sanna Toppila-Salmi

**Affiliations:** 1 Haartman Institute, University of Helsinki, Helsinki, Finland; 2 Nordic Healthcare Group, Helsinki, Finland; 3 Department of Public Health, University of Helsinki, Helsinki, Finland; 4 Department of Otorhinolaryngology-Head and Neck Surgery, Helsinki University Hospital and University of Helsinki, Helsinki, Finland; 5 Skin and Allergy Hospital, Helsinki University Hospital and University of Helsinki, Helsinki, Finland; National University of Sciences and Technology (NUST), PAKISTAN

## Abstract

**Background:**

Revision endoscopic sinus surgery (ESS) is often considered for chronic rhinosinusitis (CRS) if maximal conservative treatment and baseline ESS prove insufficient. Emerging research outlines the risk factors of revision ESS. However, accurately predicting revision ESS at the individual level remains uncertain. This study aims to examine the prediction accuracy of revision ESS and to identify the effects of risk factors at the individual level.

**Methods:**

We collected demographic and clinical variables from the electronic health records of 767 surgical CRS patients ≥16 years of age. Revision ESS was performed on 111 (14.5%) patients. The prediction accuracy of revision ESS was examined by training and validating different machine learning models, while the effects of variables were analysed using the Shapley values and partial dependence plots.

**Results:**

The logistic regression, gradient boosting and random forest classifiers performed similarly in predicting revision ESS. Area under the receiving operating characteristic curve (*AUROC*) values were 0.744, 0.741 and 0.730, respectively, using data collected from the baseline visit until six months after baseline ESS. The length of time during which data were collected improved the prediction performance. For data collection times of 0, 3, 6 and 12 months after baseline ESS, *AUROC* values for the logistic regression were 0.682, 0.715, 0.744 and 0.784, respectively. The number of visits before or after baseline ESS, the number of days from the baseline visit to the baseline ESS, patient age, CRS with nasal polyps (CRSwNP), asthma, non-steroidal anti-inflammatory drug exacerbated respiratory disease and immunodeficiency or suspicion of it all associated with revision ESS. Patient age and number of visits before baseline ESS carried non-linear effects for predictions.

**Conclusions:**

Intelligent data analysis identified important predictors of revision ESS at the individual level, such as the frequency of clinical visits, patient age, Type 2 high diseases and immunodeficiency or a suspicion of it.

## Introduction

Chronic rhinosinusitis (CRS) is a symptomatic inflammatory disease of the nasal and paranasal mucosa lasting more than 12 weeks [1]. With a prevalence of about 11%, CRS diminishes patient quality of life and productivity and increases healthcare costs [1]. The main phenotypes are CRS with nasal polyps (CRSwNP) and without (CRSsNP) [1–3]. The majority of CRS cases occurring in Western countries are characterised by Type 2 high inflammation with elevated levels of eosinophils, interleukin-4 (IL-4), IL-5 and IL-13 [1]. Nonsteroidal anti-inflammatory drug (NSAID) exacerbated respiratory disease is a Type 2 high chronic inflammatory syndrome with a partially unknown pathobiology associated with CRSwNP and asthma and with an increased morbidity [4–6].

Endoscopic sinus surgery (ESS) represents a cost-effective treatment [7] if conservative therapy (such as intranasal corticosteroids and nasal saline irrigation) is insufficient [1]. The success rates for initial ESS range from 76% to 98% [8, 9]. The early identification of CRS recurrence risk following ESS is cost-effective [10, 11], helping to correctly target treatment [12] and prevent permanent tissue changes [1].

A substantial number of studies have identified the risk factors of revision ESS [13–21], in studies varying according to sample size (n = 66 [21] vs. n = 61 000 [15]), data collection methods (large retrospective database [15] vs. prospective questionnaires [14]) or geographic location (USA [15], Australia [22] and Finland [13]). Commonly recognised risk factors include nasal polyps, asthma, allergy, non-steroidal anti-inflammatory drug (NSAID) exacerbated respiratory disease (NERD) and a previous ESS. In a meta-analysis [19], the strongest predictors of revision ESS were allergic fungal rhinosinusitis, NERD, asthma, prior polypectomy and operations prior to 2008. However, no prior research has analysed the prediction accuracy of revision ESS at the individual level or for variables with a nonlinear association. In this study, we examined the accuracy of the personalised prediction of revision ESS, and attempted to identify the effects of important predictor variables via modern machine-learning algorithms and methods.

## Materials and methods

### Patients

This study consisted of rhinitis or rhinosinusitis patients presenting at the Department of Otorhinolaryngology at the Hospital District of Helsinki and Uusimaa (HUS), Finland. The HUS ethics committee approved the study protocol (nro 31/13/03/00/2015), thereby precluding the need to obtain written informed consent from patients for this retrospective follow-up study.

The inclusion criteria for the initial patient population (*n* = 5080) was an ICD-10 diagnosis of *J*30, *J*31, *J*32, *J*33 or *J*01 registered during outpatient visits in 2005, 2007, 2009, 2011 or 2013. Longitudinal data for a random patient sample were collected from the electronic health records (EHRs), such that the sample size was the same for each sampling year and each month of the sampling year. The last data collection day for follow-up was 31 September 2019. CRS was defined as diagnostic codes *J*33 and/or *J*32. ESS was defined based on the surgical codes (Table A in S1 File). In total, we excluded 27 CRS patients <16 years of age. The baseline visit was defined as the first clinic visit, and baseline ESS was defined as the first ESS identified in EHRs at the specific sampling time. Revision ESS was defined as an ESS performed following the baseline ESS during the follow-up period.

**Fig 1:**
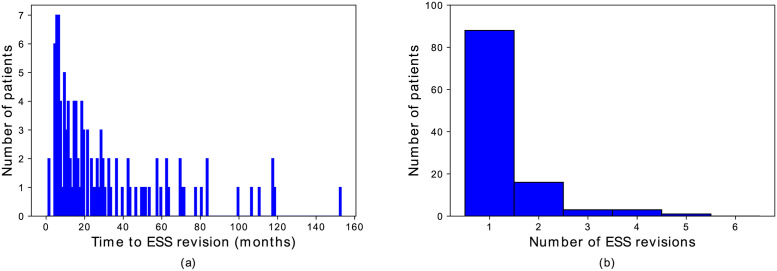
Histograms of (a) time to revision ESS and (b) the number of revision ESS surgeries. ESS, endoscopic sinus surgery.

A total of 111 of 767 (14.5%) CRS patients underwent revision ESS (mean±stdev) 30.3±31.0 months following the baseline ESS (Fig 1a and Table 1). Among revised patients, 88 underwent one revision ESS and 23 patients underwent two or more revisions (Fig 1b).

**Table 1:** Average follow-up time and number of ESS surgeries among patients with and without revision ESS.

Patients	Number of patients	Follow-up-time, Avg. (SD)	Number of ESS operations, Avg. (SD)
All	767	74.2 (42.9)	1.19 (0.54)
No revision	656	81.6 (40.2)	1.0 (0.0)
Yes revision	111	30.3 (31.0)	2.32 (0.74)

ESS = Endoscopic sinus surgery, Avg. = Average, SD = Standard deviation.

### Variables

Table 2 summarises the patient characteristics that were analysed both from the structured EHR data (visits, procedure codes and patient diagnoses) and free clinical texts (diagnoses and comorbidities). Comorbidity-related variables were obtained from the ICD-10 codes (Table B in S1 File) and using validated keyword-based information extraction from free clinical texts (see S2 File). For asthma, we used ICD-10 code *J*45, doctor-diagnosed lung function test–confirmed asthma. A NERD diagnosis was obtained from EHR text and was based on a typical history of airway symptoms following the ingestion of NSAID with/without challenge test confirmation of NERD.

**Table 2:** Characteristics of patients without and with the revision ESS. P values calculated using the Fisher’s exact or Mann-Whitney U test.

Variable	NO revision	YES revision	P value
Gender female, n (%)	382 (58.23%)	66 (59.46%)	0.836
Asthma, n (%)	230 (35.06%)	67 (60.36%)	<.001**
Allergy, n (%)	228 (34.76%)	56 (50.45%)	0.002*
Chronic respiratory diseases, n (%)	182 (27.74%)	43 (38.74%)	0.024*
Mental disorders, n (%)	79 (12.04%)	15 (13.51%)	0.64
Memory disorders, n (%)	13 (1.98%)	3 (2.7%)	0.716
Cancer, n (%)	66 (10.06%)	16 (14.41%)	0.183
Cardiovascular disease, n (%)	215 (32.77%)	47 (42.34%)	0.052
Obesity, n (%)	57 (8.69%)	8 (7.21%)	0.714
Diabetes, n (%)	63 (9.6%)	13 (11.71%)	0.493
Musculoskeletal diseases, n (%)	255 (38.87%)	50 (45.05%)	0.249
NERD, n (%)	55 (8.38%)	22 (19.82%)	<.001**
Immunodeficiency, n (%)	3 (0.46%)	2 (1.8%)	0.155
Immunodeficiency or its suspicion, n (%)	17 (2.59%)	13 (11.71%)	<.001**
Obstr sleep apnea, n (%)	56 (8.54%)	14 (12.61%)	0.21
Mouth breathing, n (%)	35 (5.34%)	11 (9.91%)	0.08
Gastroesophageal reflux, n (%)	41 (6.25%)	11 (9.91%)	0.156
CRSwNP, n (%)	204 (31.1%)	60 (54.05%)	<.001**
Age, baseline ESS, Avg. (SD)	45.61 (15.83)	48.52 (13.9)	0.028*
ASA value, Avg. (SD)	1.68 (0.63)	1.8 (0.64)	0.035*
Time from the baseline visit to the baseline ESS (days), Avg. (SD)	565.77 (824.33)	483.2 (766.65)	0.014*
Number of visits from the baseline to the baseline ESS, Avg. (SD)	4.31 (4.86)	4.97 (7.7)	0.239
Visit frequency between the baseline visit to the baseline ESS, Avg. (SD)	8.24 (19.05)	10.57 (19.02)	0.001*
Number of visits before the baseline ESS (0–12 months), Avg. (SD)	2.85 (2.51)	3.06 (3.31)	0.343
Number of visits before the baseline ESS (0–6 months), Avg. (SD)	2.07 (1.96)	2.43 (2.45)	0.186
Number of visits from the baseline ESS to 3 months postoperatively, Avg. (SD)	1.45 (1.24)	2.33 (1.98)	<.001**
Number of visits from the baseline ESS to 6 months postoperatively, Avg. (SD)	1.89 (1.81)	3.94 (3.68)	<.001**
Number of visits from the baseline ESS to 12 months postoperatively, Avg. (SD)	2.38 (2.53)	6.3 (5.55)	<.001**

ESS = Endoscopic sinus surgery, NERD = Patient-reported non-steroidal anti-inflammatory drug -exacerbated respiratory disease, CRSwNP = Chronic rhinosinusitis with nasal polyps, Avg. = Average, SD = Standard deviation.

### Machine learning algorithms

**Fig 2:**
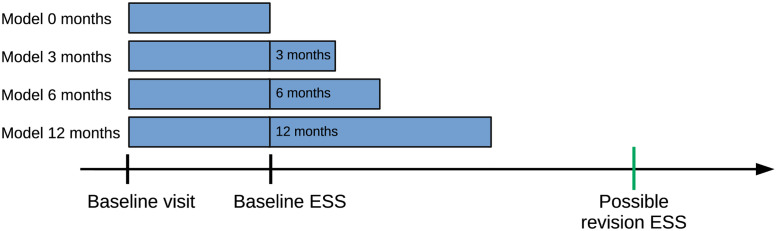
Data collection for the analysis of the effect of the data collection time period. The classifiers’ performance was calculated based on when the data were collected from the timeline of the baseline visit to the baseline ESS or to 3, 6 or 12 months following the baseline ESS. The baseline visit represents the initial clinic visit, and baseline ESS represents the first ESS identified in EHRs. Revision ESS represents the ESS performed following baseline ESS during the follow-up time period. The models were trained to predict the revision ESS. ESS, endoscopic sinus surgery; EHR, electronic health record.

In this study, we conducted four analyses: univariate model, machine learning classifier comparison, the effect of the data collection time and model interpretability analyses. The univariate models examined the prediction accuracy of individual variables using univariate logistic regression classifiers. Machine learning classifier comparison examined the predictive performance of three classifiers: random forest, logistic regression and gradient boosting. The random forest and gradient boosting classifiers were chosen for the machine learning classifier comparison since they are widely used with a demonstrated good performance [23]. Logistic regression was chosen because it is simple and still performs relatively well [24]. It remains important to determine if simpler algorithms perform comparably well. To understand the effect of the time of data collection time, the performance of the classifier was calculated when the variable collection time was from the baseline visit to the baseline ESS or to 3, 6 or 12 months following baseline ESS. Fig 2 illustrates the timeline of the data collection for the models 0, 3, 6 and 12 months, respectively. For example, the model for 3 months was trained and validated using patient data collected from patient EHRs between patients’ baseline visits and 3 months following baseline ESS. The logistic regression classifier was selected for the analysis of the data collection time period because it is simple and because the machine learning classifier study demonstrated that its performance was higher or similar to other classifiers.

However, the logistic regression classifier is linear and thus not able to model possible nonmonotonic relationships between predictors and outcomes. The random forest and gradient boosting classifiers can model complex, non-monotonous relationships, but are so-called black box models or uninterpretable classifiers. The relationships between inputs and outputs are difficult to understand directly from the parameters or the structure of the trained model. For the model interpretability analysis, we chose to use gradient boosting classifiers. The model interpretability analysis was calculated using Shapley values (SHAP) and partial dependence plots (PDPs) [25, 26], and were analysed for their importance and the possible nonmonotonic effects of the variables.

### Model training

**Fig 3:**
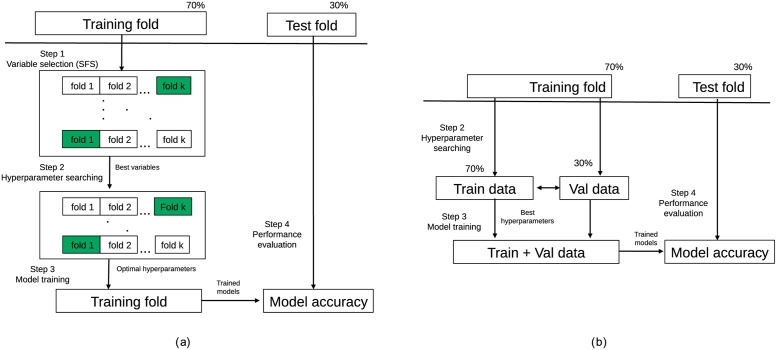
Data flows for the prediction model learning pipelines. Variable selection (step 1), search for hyperparameters (step 2), model training (step 3) and performance evaluation (step 4). Data flow (a) is used to compare machine learning classifiers and the effect of data collection time period analyses. Data flow (b) is used for the univariate model and model interpretability analyses.

Fig 3 shows the data flows for training and testing the classifiers. Original data were first divided into two distinct data folds: the training fold (70% of the data) and the test fold (30% of the data). We used the training fold to select the variables and hyperparameters and to train the final models. The test fold relied on an external dataset which we used to measure the performance of the final models. During splitting, folds were stratified to preserve the proportion of patients in both target classes (no revision vs. revision).

The data flow shown in Fig 3a was used to compare the machine learning classifiers and for the analysis of the data collection time period. Here, we summarise the steps in the process, which included variable selection (step 1), searching for model hyperparameters (step 2), model training (step 3) and performance evaluation (step 4). The data flow in Fig 3b provides the univariate model and the model interpretability analyses. Specifically, we proceeded by searching for model hyperparameters (step 2), model training (step 3) and performance evaluation (step 4). One primary difference between the data flows presented is that Fig 3a uses the k-fold cross-validation for variable selection (SFS, sequential forward selection). Data flow of Fig 3b relies on predefined variables for training models.

### Sequential forward variable selection

The data flow in Fig 3a contains the method of sequential forward variable selection (SFS, step 1) [27]. SFS begins with an empty set, and adds one variable at a time from the original variable set **S**_*all*_ (Table 2) for classifier *F*(⋅) by maximising the performance measure. We used the area under the receiver operating characteristic (*AUROC*) curve as the performance metric. Because our data are unbalanced, we used class weight balanced loss functions. The output of the SFS was 15 most important variables **S**_*k*,*sel*_ for classifier *F*(⋅). The (average) importance of each variable *a* was measured using the following rank metric:

$$\begin{array}{c} R\left(a\right)=\frac{1}{10}\sum_{k=1}^{10}\left(\#F-r\left(k,a\right)+1\right)¸ \end{array}$$ (1)

where *r*(*k*, *a*) is the rank of variable *a* based on the data set *k* and #*F* is the size of the largest subset resulting from SFS [28–30]. In this study, #*F* = 15. A higher *R*(*a*) (rank score) indicates that variable *a* is more important according to SFS, because it was selected in the smaller size variable subsets. That is, the variable has a higher predictive capability according to SFS, whereby its revision ESS prediction ability is high. The optimal hyperparameters for classifier *F*(⋅) with variables **S**_*m*,*sel*_ were identified using the grid-search method (step 2). The hyperparameter values for different classifiers and the summary statistics for the selected hyperparameter values appear in S5 File. Following the identification of the optimal hyperparameters for classifier *F*(⋅) using variables **S**_*m*,*sel*_, the model was trained (step 3) and the performance was calculated using dataset **X**_*test*_ (step 4).

### Performance analysis

We used the following standard performance metrics: *AUROC*, the area under the precision recall curve (*AUPRC*), precision, sensitivity, specificity and the F1 score. *AUROC* is the mostly used evaluation metric for measuring the performance of any classification model. An *AUROC* of 0.5 indicates no discrimination above chance, while an *AUROC* of 1.0 indicates a perfect classification. A rough guide for the classification ability of a model is *AUROC* = 0.9–1.0 indicates an excellent performance, *AUROC* = 0.8–0.9 indicates a good performance, *AUROC* = 0.7–0.8 indicates a fair performance and *AUROC* = 0.6–0.7 indicates a poor performance [31, 32]. *AUPRC* is often used evaluation metric for imbalanced data sets. The baseline (discrimination above change) of *AUPRC* is equal to the fraction of positives. The baseline *AUPRC* of our study is 0.145, indicating that 14.5% of the patients underwent revision ESS. The baseline values for the AUROC and AUPRC metrics were confirmed for our data by training and testing the models using randomised label data (Table A in S3 File).

Precision refers to the number of true positive results divided by the number of all positive results, including those not identified correctly. In this study, precision refers specifically to the ability of a model to identify only revision patients. Sensitivity, by comparison, indicates the number of true positive results divided by the number of all samples that should have been identified as positive. In this study, then, sensitivity refers specifically to the ability of the model to identify all of the revision patients. Specificity is the number of true negative results divided by the number of all samples that should have been identified as negative. In this study, specificity specifically refers to the ability of the model to identify all patients not needing revision. Finally, the F1 score represents the harmonic mean between the precision and sensitivity. Precision, sensitivity, specificity and F1 score are calculated using the following equations:

$$\begin{array}{c} Precision=\frac{TP}{TP+FP} \end{array}$$ (2)

$$\begin{array}{c} Sensitivity=\frac{TP}{TP+FN} \end{array}$$ (3)

$$\begin{array}{c} Specificity=\frac{TN}{TN+FP} \end{array}$$ (4)

$$\begin{array}{c} F1-score=\frac{Precision*Sensitivity}{Precision+Sensitivity} \end{array}$$ (5)

where *TP* is the number of true positives predicted by the classifier, *FP* is the number of false positives, *FN* is the number of false negatives and *TN* is the number of true negatives.

### Software

We used seven Python packages—*sklearn* [33], *xgboost* [34], *mlxtend* [35], *numpy* [36], *pandas* [37], *shap* [25, 38] and *pdpbox* [39]—to implement the classifiers and compute the performance values and model interpretations. SFS was computed using the ‘SequentialFeatureSelector’ function in the mlxtend package. The classifiers of random forest, logistic regression and gradient boosting were implemented using functions from the *sklearn*.*linear*_*model*, *sklearn*.*ensemble* and *xgboost* packages. The grid search for the hyperparameters was conducted using the ‘GridSearchCV’ function in the *sklearn*.*model*_*selection* package. We computed the Shapley values using the ‘TreeExplainer’ function in the shap package. Partial dependency plots (PDPs) were created using the ‘pdp_isolate’ function in the *pdpbox* package. The packages of *numpy* and *pandas* were used for data reading and processing.

## Results

The CRS patient population which underwent baseline ESS (*n* = 767), included 448 (58%) females, ranging in age from 16 to 90 years. Table 2 summarises the patient characteristics and the proportion who did and did not undergo revision ESS. The following comorbidities significantly associated with patients who underwent revision ESS during follow-up: doctor-diagnosed lung function test-confirmed asthma, CRSwNP, allergies, chronic respiratory disease, EHR text-based NERD and immunodeficiency or a suspicion of immunodeficiency. The following continuous variables significantly associated with revision ESS: an older age, a shorter time from the baseline visit to baseline ESS, a higher frequency of visits between the baseline visit and baseline ESS and a higher number of visits from the baseline ESS to 3 months postoperatively, 6 months postoperatively and 12 months postoperatively, respectively.

### Univariate analyses

Table 3 presents the results of the univariate logistic regression models to predict revision ESS following baseline ESS. Results represent the average of 10 reformulations from the training and test folds (Fig 3b). Among continuous variables, the highest AUROC values were for the number of visits 12, 6, and 3 months following baseline ESS (*AUROC* = 0.77, 0.70, 0.66, respectively). The next highest AUROC values were for the time between the baseline visit and baseline ESS (*AUROC* = 0.59) and for the frequency of visits between the baseline visit and baseline ESS (*AUROC* = 0.58). Among categorical variables, the highest AUROC values were for asthma (*AUROC* = 0.65), CRSwNP (*AUROC* = 0.64), immunodeficiency or a suspicion of it (*AUROC* = 0.61), allergies (*AUROC* = 0.60), chronic respiratory diseases (*AUROC* = 0.59) and NERD (*AUROC* = 0.59). We also found that the odds ratios (ORs) for continuous variables all exceeded 1.0 with one exception, indicating that a higher number of visits and greater frequency of visits and a shorter time between baseline visit and baseline ESS increased the probability of a revision ESS.

**Table 3:** Odds ratios (ORs) and performance values (AUROC, sensitivity, specificity and F1 score) for predicting a revision ESS using different variables in univariate logistic regression models.

Variable	OR (95% CI)	AUROC (95% CI)	Sensitivity (Avg)	Specificity (Avg)	F1 score (Avg)
Number of visits from the baseline ESS to 12 months postoperatively	112.62 (105.62–120.09)	0.77 (0.76–0.78)	0.58	0.80	0.43
Number of visits from the baseline ESS to 6 months postoperatively	38.3 (36.03–40.71)	0.7 (0.69–0.7)	0.55	0.72	0.34
Number of visits from the baseline ESS to 3 months postoperatively	10.18 (9.58–10.81)	0.66 (0.65–0.66)	0.54	0.65	0.30
Asthma	2.51 (2.44–2.59)	0.65 (0.64–0.66)	0.60	0.65	0.32
CRSwNP	2.61 (2.54–2.68)	0.64 (0.63–0.65)	0.56	0.64	0.31
Immunodeficiency or its suspicion	3.46 (3.32–3.62)	0.61 (0.6–0.61)	0.50	0.63	0.28
Allergy	1.81 (1.76–1.86)	0.6 (0.59–0.61)	0.55	0.58	0.27
Chronic respiratory diseases	1.49 (1.45–1.53)	0.59 (0.58–0.6)	0.57	0.57	0.27
NERD	2.4 (2.33–2.48)	0.59 (0.58–0.6)	0.45	0.63	0.26
Obstr sleep apnea	1.37 (1.32–1.43)	0.58 (0.57–0.59)	0.55	0.55	0.26
Visit frequency between the baseline visit to the baseline ESS	1.43 (1.32–1.55)	0.58 (0.57–0.58)	0.56	0.55	0.26
Time from the baseline visit to the baseline ESS (days)	0.43 (0.4–0.46)	0.58 (0.57–0.58)	0.55	0.57	0.27
Age, baseline ESS	2.12 (1.99–2.25)	0.58 (0.57–0.58)	0.54	0.55	0.26
Mouth breathing	1.59 (1.51–1.68)	0.58 (0.57–0.59)	0.56	0.56	0.27
Immunodeficiency	1.53 (1.42–1.66)	0.58 (0.57–0.58)	0.56	0.55	0.27
Cardiovascular disease	1.33 (1.29–1.37)	0.58 (0.57–0.59)	0.52	0.56	0.26
Gastroesophageal reflux	1.4 (1.34–1.46)	0.57 (0.56–0.58)	0.53	0.55	0.26
Cancer	1.18 (1.14–1.23)	0.57 (0.56–0.58)	0.53	0.55	0.26
Gender female	0.99 (0.97–1.02)	0.57 (0.56–0.57)	0.55	0.55	0.26
Musculoskeletal diseases	1.14 (1.11–1.18)	0.57 (0.56–0.57)	0.52	0.54	0.24
ASA value	1.43 (1.38–1.49)	0.57 (0.56–0.58)	0.54	0.55	0.26
Diabetes	1.05 (1.01–1.09)	0.57 (0.56–0.58)	0.55	0.54	0.26
Number of visits from the baseline to the baseline ESS	1.1 (1.03–1.17)	0.57 (0.56–0.58)	0.55	0.55	0.26
Obesity	0.72 (0.68–0.77)	0.57 (0.56–0.58)	0.55	0.55	0.26
Number of visits before the baseline ESS (0–12 months)	1.01 (0.94–1.09)	0.57 (0.56–0.58)	0.55	0.55	0.26
Number of visits before the baseline ESS (0–6 months)	1.73 (1.6–1.87)	0.57 (0.57–0.58)	0.53	0.56	0.26
Mental disorders	0.92 (0.89–0.96)	0.57 (0.56–0.57)	0.53	0.55	0.26
Memory disorders	1.06 (0.98–1.15)	0.57 (0.56–0.58)	0.55	0.54	0.26

ESS = Endoscopic sinus surgery, OR = Odds ratio, 95% CI = 95% Confidence interval, AUROC = Area under the receiver operating characteristics curve, Avg. = Average, F1-score = Harmonic mean between precision and sensitivity, CRSwNP = Chronic rhinosinusitis with nasal polyps, NERD = Non-steroidal anti-inflammatory drug –exacerbated respiratory disease, ASA-value = American Society of Anesthesiology score (= a metric to determine if someone is healthy enough to tolerate surgery and anesthesia).

### Machine learning classifier comparison

**Fig 4:**
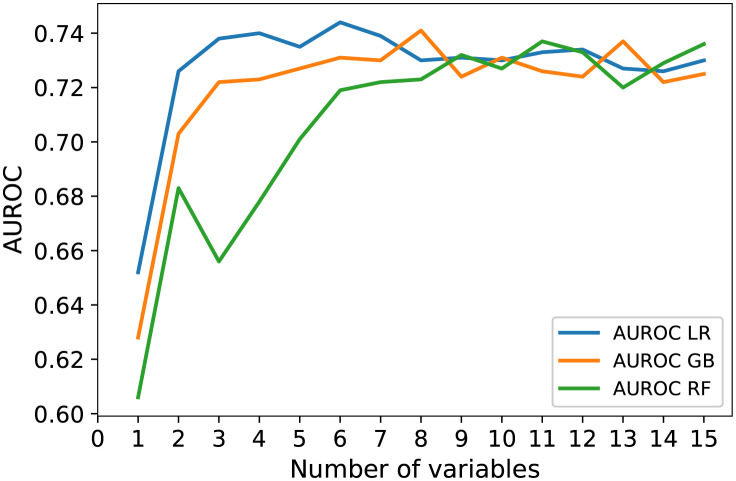
AUROC values as a function of the number of variables used to predict revision ESS. We used three models and the classifiers in these models were logistic regression, gradient boosting and random forest for predicting revision ESS. AUROC, area under the receiver operating characteristics curve; ESS, endoscopic sinus surgery.

The plots in Fig 4 show the *AUROC* values for the classifiers of random forest, logistic regression and gradient boosting as a function of the number of variables. We applied the SFS method to select variables collected from the baseline visit until six months after the baseline ESS. Results are reported as the averages from 10 reformulations from the training and test folds (see Fig 3a). The *AUROC* values first increased rapidly and then reached a plateau as a function of the number of variables. For the logistic regression classifier, the highest average *AUROC* (0.744) was achieved using six variables. For the gradient boosting classifier, the highest *AUROC* (0.741) was with eight variables. For the random forest classifier, the highest *AUROC* (0.737) was with 11 variables. The *AUPRC* values for the same number of variables were 0.354, 0.348 and 0.378, respectively. The baseline *AUROC* and *AUPRC* values (presenting discrimination above chance) calculated using the random classifier were 0.499, 0.472 0.489 and 0.149, 0.151 0.149 (Table A in S3 File). The performance of the ensemble model in which the logistic regression, random forest and gradient boosting classifiers were combined was comparable with the performance of each individual classifier (Table A in S4 File). Tables 4 and 5 summarise the *AUROC*, *AUPRC*, sensitivity and specificity values as a function of the number of variables.

**Table 4:** AUROC and AUPRC values as a function of the number of variables for predicting revision ESS. We selected variables using the sequential forward selection (SFS) method. Three models were used and the classifiers in these models were the logistic regression (LR), gradient boosting (GB) and random forest (RF) for predicting revision ESS. Table 4 (AUROC values) is related to Fig 4.

Number of variables	AUROC LR	AUROC GB	AUROC RF	AUPRC LR	AUPRC GB	AUPRC RF
1	0.652	0.628	0.606	0.327	0.322	0.317
2	0.726	0.703	0.683	0.332	0.345	0.326
3	0.738	0.722	0.656	0.341	0.341	0.298
4	0.740	0.723	0.678	0.350	0.356	0.312
5	0.735	0.727	0.701	0.347	0.368	0.318
6	0.744	0.731	0.719	0.354	0.360	0.319
7	0.739	0.730	0.722	0.347	0.352	0.339
8	0.730	0.741	0.723	0.344	0.378	0.334
9	0.731	0.724	0.732	0.343	0.351	0.352
10	0.730	0.731	0.727	0.343	0.365	0.339
11	0.733	0.726	0.737	0.345	0.351	0.348
12	0.734	0.724	0.733	0.338	0.360	0.349
13	0.727	0.737	0.720	0.331	0.366	0.347
14	0.726	0.722	0.729	0.334	0.349	0.342
15	0.730	0.725	0.736	0.336	0.343	0.337

AUROC = Area under the receiver operating characteristics curve, AUPRC = Area under the precision recall curve, ESS = Endoscopic sinus surgery.

**Table 5:** Sensitivity and specificity values as a function of the number of variables for predicting revision ESS. We selected variables using the sequential forward selection (SFS) method. Three models were used and the classifiers in these models were the logistic regression (LR), gradient boosting (GB) and random forest (RF) for predicting revision ESS.

Number of variables	Sensitivity LR	Sensitivity GB	Sensitivity RF	Specificity LR	Specificity GB	Specificity RF
1	0.455	0.306	0.370	0.795	0.914	0.825
2	0.621	0.330	0.542	0.693	0.921	0.696
3	0.609	0.373	0.527	0.745	0.919	0.686
4	0.621	0.427	0.518	0.759	0.887	0.745
5	0.636	0.400	0.485	0.752	0.907	0.781
6	0.618	0.427	0.485	0.752	0.884	0.794
7	0.618	0.352	0.485	0.756	0.915	0.817
8	0.615	0.418	0.458	0.757	0.899	0.826
9	0.618	0.391	0.512	0.753	0.896	0.796
10	0.621	0.394	0.479	0.750	0.905	0.826
11	0.615	0.364	0.455	0.757	0.917	0.820
12	0.579	0.433	0.442	0.760	0.886	0.832
13	0.567	0.355	0.476	0.765	0.914	0.818
14	0.567	0.367	0.445	0.763	0.912	0.822
15	0.573	0.373	0.461	0.764	0.896	0.835

ESS = endoscopic sinus surgery.

Table 6 presents the variables selected using SFS in order of the rank scores calculated using Eq 1. When using any of the three classifiers, the following variables resulted in high rank scores, indicating their importance as predictors of revision ESS: the number of visits six months after baseline ESS, CRSwNP, asthma and NERD. In addition, the frequency of visits from the baseline visit to baseline ESS and the number of visits before the baseline ESS emerged as important predictors.

**Table 6:** The top ten variables in prediction capacity of revision ESS. Three models were used and the classifiers of the three models were the logistic regression, gradient boosting and random forest for predicting revision ESS. We used the sequential forward selection (SFS) method to select the top performing variables. In each SFS run, the best variable was awarded 15 points, the next best variable 14 points and so on. Ten runs were performed using each of the three classifiers. The rank score represents the sum of points (range, 0–150 points).

Classifier	Variables	Rank score
Logistic regression	Number of visits from the baseline ESS to 6 months postoperatively	148
	CRSwNP	112
	Asthma	111
	Immunodeficiency or its suspicion	87
	NERD	63
	Number of visits before the baseline ESS (0–12 months)	55
	Visit frequency between the baseline visit to the baseline ESS	52
	Age, baseline ESS	49
	Gastroesophageal reflux	46
	Number of visits from the baseline ESS to 3 months postoperatively	41
Gradient boosting	Number of visits from the baseline ESS to 6 months postoperatively	149
	CRSwNP	134
	Asthma	100
	Memory disorders	98
	Obesity	80
	Number of visits before the baseline ESS (0–12 months)	78
	NERD	58
	Immunodeficiency or its suspicion	52
	Mental disorders	47
	Immunodeficiency	37
Random forest	Number of visits from the baseline ESS to 6 months postoperatively	148
	Asthma	122
	CRSwNP	90
	Visit frequency between the baseline visit to the baseline ESS	72
	Age, baseline ESS	70
	Immunodeficiency or its suspicion	68
	Diabetes	68
	Memory disorders	63
	Cancer	55
	NERD	53

AUROC = Area under the receiver operating characteristics curve, ESS = Endoscopic sinus surgery, CRSwNP = Chronic rhinosinusitis with nasal polyps, NERD = Non-steroidal anti-inflammatory drug –exacerbated respiratory disease.

### Effect of the data collection time

**Fig 5:**
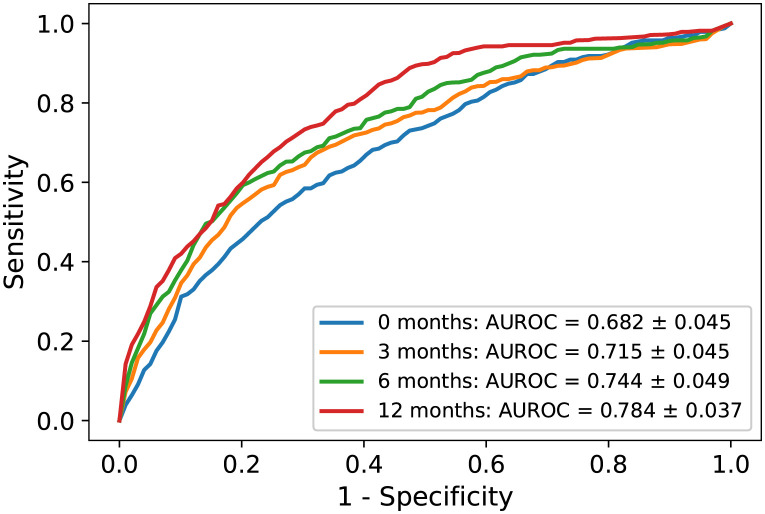
Receiver operating characteristics curves (ROC) to predict revision ESS. We used the logistic regression classifier in the four models, which used four different data collection time periods. The data collection time periods (0, 3, 6 or 12 months) indicate the times during which data were collected after baseline ESS. ESS, endoscopic sinus surgery.

The effect of the length of time for data collection on the model’s ability to predict the risk of revision ESS was evaluated using the logistic regression classifier. Fig 5 presents the AUROC values when the data collection time period was from the baseline visit to the baseline ESS or until 3, 6 or 12 months after baseline ESS. Table 7 summarises the *AUROC*, *AUPRC*, sensitivity, specificity and F1 score values. The highest performance (*AUROC* = 0.784) was, as expected, found in the model that included a 12-month follow-up period, because more information was available in that model compared with models using 3- or 6-month follow-up periods or no follow-up period at all. The sensitivity for the 12-month model reached 0.61, indicating that the model identified 61% of patients needing revision ESS. The specificity for the 12-month model reached 0.79, indicating that 79% of patients classified as negative did not need revision ESS.

**Table 7:** Average performance values using all of the variables to predict revision ESS by using different data collection time periods in the logistic regression models. The data collection time periods (0, 3, 6 or 12 months) indicate the time during which data were collected after baseline ESS. See also Fig 5.

Model	AUROC (95% CI)	AUPR (95% CI)	Sensitivity (95% CI)	Specificity (95% CI)	F1-score (95% CI)
0 months	0.682 (0.648–0.716)	0.259 (0.231–0.287)	0.552 (0.48–0.623)	0.714 (0.677–0.751)	0.337 (0.307–0.367)
3 months	0.715 (0.681–0.749)	0.316 (0.271–0.361)	0.606 (0.538–0.675)	0.732 (0.702–0.763)	0.377 (0.345–0.409)
6 months	0.744 (0.707–0.781)	0.354 (0.303–0.404)	0.618 (0.561–0.675)	0.752 (0.73–0.773)	0.398 (0.367–0.429)
12 months	0.784 (0.756–0.813)	0.411 (0.352–0.471)	0.606 (0.547–0.665)	0.787 (0.762–0.813)	0.422 (0.379–0.464)

ESS = Endoscopic sinus surgery, 95% CI = 95% Confidence interval, AUROC = Area under the receiver operating characteristics curve, AUPRC = Area under the precision recall curve, F1-score is the harmonic mean between precision and sensitivity.

### Interpretability analysis

**Fig 6:**
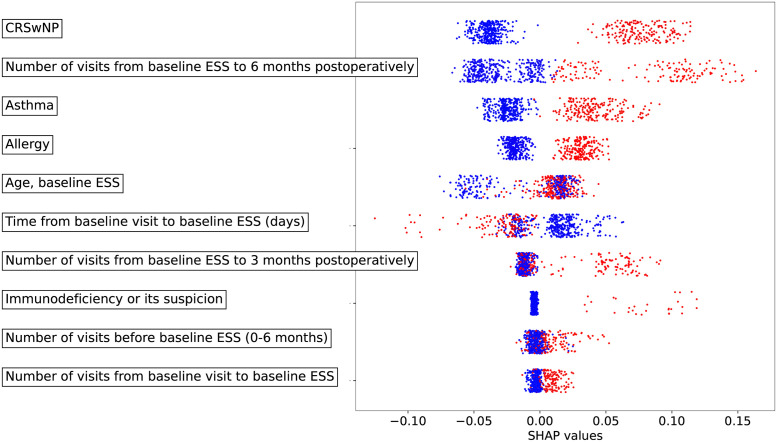
Shapley values (SHapley Additive exPlanations, SHAP) for the ten most important variables predicting revision ESS. The gradient boosting algorithm was used as the classifier. The definition of the ten most important variables is based on the sum of the absolute Shapley values. The red points indicate higher patient-specific variable values than the average value of the variable and, the blue points indicate lower patient-specific variable values than the average value for the variable. A longer distance between a red and blue point indicates a better capacity of the variable to predict revision ESS. ESS, endoscopic sinus surgery; CRSwNP, chronic rhinosinusitis with nasal polopys.

For the model interpretability analysis, we trained the gradient boosting classifier using the variables collected from the baseline visit until 6 months following baseline ESS employing the data flow from Fig 3b. Fig 6 illustrates the variables sorted based on the highest sum from the absolute Shapley values across all patients. The distributions of the data points on the plots show the impact of each variable on the classifier output. We found that a high number of visits after baseline ESS and a short time interval between the baseline visit and baseline ESS both increased the revision ESS risk. In addition, CRSwNP, asthma and allergies increased the revision ESS risk. The Shapley values revealed that patient age and the frequency of clinical visits from baseline visit to baseline ESS (that is, the time period from the baseline visit to the baseline ESS and the number of visits before baseline ESS) affected the revision ESS risk in a nonmonotonic manner. That is, the red values (the higher than the average values) of these variables are dispersed on both sides of the scale.

**Fig 7:**
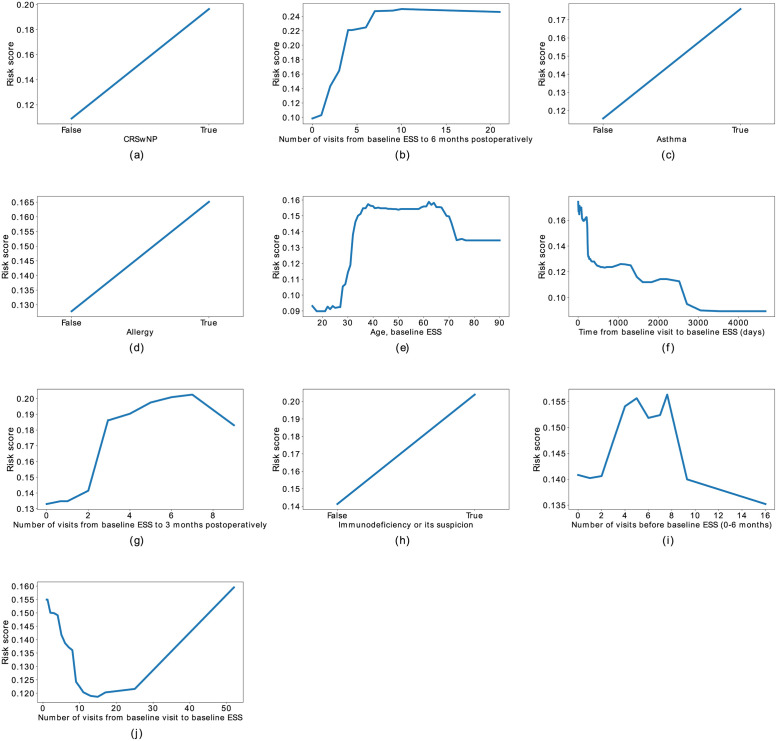
Partial dependence plots (PDP) for predicting revision ESS using the gradient boosting classifier of the ten variables with the highest SHAP values (Shapley or SHapley Additive exPlanations). ESS, endoscopic sinus surgery; CRSwNP, chronic rhinosinusitis with nasal polyps. See also Fig 6.

Fig 7 shows the PDPs for the ten variables with the highest Shapley values. The PDP plot for the number of visits 6 months following baseline ESS revealed a wide risk score range, from a value of 0.1 for patients with less than two visits following baseline ESS up to a value of about 0.26 for patients with more than seven visits (Fig 7b). Similarly, if the patient had two or more postoperative visits within 3 months, the risk score for revision ESS increased (Fig 7g). The plot for the time between the baseline visit and baseline ESS revealed a sharp drop in the risk score after about 100 days (Fig 7f). When the time between the baseline visit and ESS was less than 100 days, the risk score was about 0.15. When the time increase to >500 days, the risk score decreased to <0.13. The PDP curve for age was nonmonotonic and the risk scores varied from 0.1 for patients aged 16 to 30 years to about 0.14 for patients aged 70 to 90 years (Fig 7e). The risk score was 0.16 for patients aged 30 to 65 years. Furthermore, the number of visits between the baseline visit and baseline ESS was nonmonotonic. Patients with 10 to 25 visits between the baseline visit and baseline ESS exhibited a smaller risk for revision ESS than patients with fewer than 10 visits or for patients with more than 25 visits (Fig 7j).

We also detected a moderate correlation between the number of days from the baseline visit to the baseline ESS and the number of visits (*p* < 0.001, correlation coefficient *r* = 0.51 from the Pearson’s linear correlation test). Yet, the correlation was weak between the number of days from the baseline visit to the baseline ESS and the following variables: age (*r* = 0.14), CRSwNP (*r* = 0.06), asthma (*r* = 0.19) and immunodeficiency (*r* = −0.00).

## Discussion

This study aimed to identify individual-level risk factors associated with revision ESS among CRS patients through the use of machine-learning algorithms. Personalised risk assessment is a process whereby an individual’s level of risk is calculated using multiple predictors [40]. Personalised risk communication represents a process through which the results of an individual’s risk assessment are tailored to their preferences and for specific uses [40]. In part, we identified previously unpublished important predictors of revision ESS, such as a high number of visits before and after baseline ESS as well as a short time interval between the baseline visit and baseline ESS. Our data also demonstrated that demographic variables such as age, Type 2 high diseases (CRSwNP, asthma and NERD) and immunodeficiency or a suspicion of it were important predictors of revision ESS at the individual level. These findings agree with previous observations at the population level [41]. In addition, our findings reinforce the importance of diagnostics and the management of NERD, nasal polyps, asthma and other comorbidities in preventing uncontrolled CRS.

In terms of clinical implications, our findings may prove relevant to patient counselling, following up on and planning treatment, such as that of biological therapy [12]. However, validation studies for these results remain necessary. Personalised risk communication has previously proven effective in clinical decision-making, such as in COVID-19 diagnostics [42], patient selection for cardiac resynchronisation therapy [43] and in organising follow-up for patients receiving adjuvant endocrine therapy [44].

To our knowledge, machine learning models have not been previously used to predict revision ESS among CRS patients. Machine learning, however, has previously been used in allergology and related research [45], including in the prediction of persistent early childhood asthma [46], eosinophilic esophagitis [47], eosinophilic CRS [48] or osteomeatal complex inflammation [49]. In addition, machine learning has found applications in predicting postoperative outcomes for degenerative cervical myelopathy [50], revision surgery following knee replacement [51], prolonged opioid prescription following surgery for lumbar disc herniation [52], blood transfusion following adult spinal deformity surgery [53], surgical infections [54] and olfactory recovery after ESS [55]. None of these previous studies, however, have presented models designed to predict revision ESS at the individual level. Revision ESS risk has previously been studied at the population level relying instead on traditional statistical models such as Cox’s proportional hazard models [13, 15, 16] or logistic regression models [14, 15, 18, 20]. Such studies have assumed associations are linear and that an alpha error <5% indicates the importance of a predictor.

We found that a greater number of visits, a higher frequency of visits and a shorter time period between the baseline visit and baseline ESS all associated with revision ESS. This might reflect that patients with a high number of visits exhibit more uncontrolled CRS and thus may ultimately undergo revision ESS. In other words, our findings suggest that an increasing number of visits before ESS might signal more severe disease, which affects not just the physician’s and but also the patient’s decision regarding ESS at baseline as well as the revision ESS during follow-up. These results indicate that patients who achieved control of disease following baseline ESS did not require further follow-up visits through tertiary care centres and were unsubscribed from the hospital. Patients with ongoing problems, however, tend to visit the clinic more frequently and exhibit a higher probability of ultimately undergoing revision ESS. We found little evidence in the literature on the predictive potential of visits at the individual level. A retrospective cohort study from the USA (*n* = 6985) revealed that the number of postoperative outpatient visits associated with revision surgery for anterior cruciate ligament reconstructions [56], findings similar to ours, albeit different types of surgeries and at a population level. Our findings indicate that patients with a higher frequency of visits at baseline exhibit a higher risk only partially controlled by surgery might prove helpful when counselling patients.

Our study also showed that CRSwNP, asthma and NERD represent important predictors of revision ESS at the individual level. In accordance with this, previous studies demonstrated at the hospital population level that several factors associate with CRS recurrence and/or revision ESS, including CRSwNP, asthma, allergic rhinitis, NERD, eosinophilia and smoking [1, 13, 57, 58]. CRSwNP patients with a comorbidity of asthma and/or NERD carry an increased risk for recurrence and revision ESS, although these patients appear to benefit from an initial ESS [19, 41, 59–61]. This finding may reflect more severe disease, typically presenting with comorbidities for NERD, anosmia, Type 2 high eosinophilic inflammation and a higher likelihood of polyp regrowth [5, 57, 62–70]. In SFS, immunodeficiency or a suspicion of it also emerged as one of the top ten predictors in all three classifiers. Immunodeficiency increases the risk of infectious exacerbations and uncontrolled CRS, thereby also increasing the risk of revision ESS. This agrees with a previous study that demonstrated that at the hospital population level immunodeficiency and granulomatosis with polyangiitis increase the revision ESS risk [71]. While the variable ‘suspicion of immunodeficiency’ is not the same as a diagnosed immunodeficiency, it might indirectly reflect a similar situation regarding poor CRS control, leaving a physician to suspect a rare comorbidity or allowing consideration for the need of revision ESS.

We also demonstrated that the length of time for EHR data collection increased the predictive accuracy of the models. The time period for data collection from the baseline visit until 12 months following the baseline ESS carried the highest predictive accuracy in our models. The time interval for data collection for the model serves to optimise the time required following baseline ESS and model accuracy.

We validated the predictive accuracy using three classifiers. To do, we chose to use logistic regression, gradient boosting and random forest classifiers since they possess different properties and generally have been used in predicting surgical outcomes [50, 72] or persistent asthma [46]. The logistic regression classifier is linear and thus incapable of modelling possible nonmonotonic and nonlinear relationships between predictors and outcomes [73]. The random forest and gradient boosting classifiers can model complex relationships, but they represent so-called black box models, meaning that are uninterpretable classifiers, whereby the relationships between inputs and results are difficult to directly interpret beyond the parameters or the structure of the trained model [73]. Since the predictive accuracy of the variables was similar across the three classifiers in our study, we used logistic regression primarily to validate the variable collection time period. Overall, our findings indicate the importance of validating outcome prediction using different classifiers and evaluating the effect of the data collection time period, as suggested in previous studies [74, 75]. By evaluating different classifiers, we found that a simple and interpretable logistic regression model may prove adequate for clinical application. However, if modelling requires nonlinear relationships, then random forest or gradient boosting models can be used. Classification performance proved comparable across all classifiers.

Revision ESS risk was previously studied at the population level using Cox’s proportional hazard [13, 15, 16] or logistic regression [14, 15, 18, 20] models, which usually assume associations are linear and that an alpha error <5% indicates the importance of a predictor. Using these assumptions, previous studies have demonstrated that a younger age associated with revision ESS [13, 66]. We found that age actually affects revision ESS risk in a nonmonotonic manner, thus indicating that machine learning improves the prediction potential of age in revision ESS risk. Similarly, nonlinear approaches have significantly improved the prediction of stroke risk [76].

Both our own and previous study groups have examined populations of CRSwNP [66] or CRS [13] patients. In our study, we found that age actually affects revision ESS risk in a nonmonotonic manner. Thus logistic regression models appear less than ideal for examining the impact of the individual patient’s age on revision ESS risk. By performing partial dependency plot analyses, we showed that the revision ESS risk was highest for patients aged 30 to 70 years, and medium high for patients older than 70 years, whereas the risk was lowest among patients aged 16 to 30 years. Younger patients experience less CRSwNP or CRSwNP among such patients often comprises antrochoanal polyps, which carry a smaller revision surgery risk [1].

Furthermore, the number of visits before baseline ESS carried nonlinear effects as predictors in our study. Patients logging 10 to 25 clinical visits between the baseline visit and baseline ESS exhibited a lower risk for revision ESS than patients with fewer than 10 or more than 25 clinic visits. Those patients visiting the clinic 10 to 25 times before baseline ESS may have CRSsNP with acute recurrent exacerbations. However, this subgroup warrants further study in order to confirm this assumption, since the number of subjects in our study was rather small. We can speculate that some physicians may schedule more frequent follow-up visits even with sufficient disease control. That said, consistent practices have been employed in our hospital, the clinical visit frequencies are closely monitored and routine controls are not reserved. Thus we argue that a visit frequency ≥2 per year reflects relatively poor disease control. Previous studies found that CRSwNP patients with recurrent acute rhinosinusitis episodes benefit from an initial ESS [1]. Previous studies on other conditions and on other predictors revealed a U-shaped association between the predictor variable and outcome, including associations between intraoperative net fluid balance and early atrial tachyarrhythmia recurrence [77] as well as between body mass index and asthma in Japanese children [78]. These findings highlight the importance of evaluating the linearity of associations to improve the personalised predictive value of them.

The strengths of this study include the random sample of hospital patients, the long follow-up time period we captured and the discovery of nonlinear associations between certain variables and outcomes. In addition, the novelty of this study lies in the validation of models employing several classifiers, which were also tested at the individual level.

We should also mention several limitations to our study, which include changes which occurred in ESS and CRS care during the sampling time period. To minimise the impact of any possible chronological or seasonal bias, we spread the sampling time over several baseline years (2005, 2007, 2009, 2011 and 2013) and each month during the baseline year. Patients with recurrence may have sought treatment elsewhere, although this potential bias was minimal since over 90% of ESS are performed in public healthcare settings [79]. In this study, we were authorised to extract data from a relatively small number of patients. However, this limitation was addressed by using cross-validation methods. Unfortunately, we did not process time series variables. Thus, recurrent neural network type models such as long short-term memory (LSTM) or bidirectional LSTM could not be used to predict revision ESS risk. EHR data have been available in our hospital since 2005. We acknowledge that the baseline ESS does not always indicate the first ESS. As such, we lacked data for possible earlier ESS, which we have previously shown to affect the revision ESS risk on a population level [13]. Furthermore, data were lacking for some other important factors, such as postoperative treatment, validated symptoms, endoscopic nasal polyp score, medication, the Lund Mackay score for sinus computed tomography scans, smoking status, eosinophils and the extent of baseline ESS. Yet, some of these variables, such as smoking [13] or total ethmoidectomy, have not emerged as strong predictors of revision ESS compared with Type 2 high diseases [57] in our previous studies. That said, we acknowledge that the inclusion of more variables and additional cases would most likely improve our estimates. Therefore, before extrapolating our results to clinical practice, replication studies in other populations and with additional variables are needed.

## Conclusions

Our results indicate that Type 2-high conditions (CRSwNP, asthma and NERD), a high clinical visit frequency, a short time interval between the baseline clinic visit and ESS and immunodeficiency or a suspicion of it increase the likelihood of revision ESS at the individual level. Moreover, age and the number of preoperative clinical visits predict a nonlinear revision ESS risk. Although these findings require validation in other populations, our results reinforce the importance of diagnostics and the management of NERD, CRSwNP, asthma and other comorbidities to prevent uncontrolled CRS, and carry relevancy for patient counselling specifically.

## Supporting information

S1 File Procedure and ICD10-codes. List of procedure and ICD10 codes that were used for identifying ESS patients and chronic diseases.
(PDF)

S2 File Key words mining from clinical texts. Keyword-based information extraction method was used for processing variables from free clinical texts.
(PDF)

S3 File Performance of baseline machine learning classifier. Baseline performance values for machine learning classifiers are presented. The values were calculated by training and testing the classifiers when the labels of data were randomized.
(PDF)

S4 File Performance of ensemble classifier. Performance values for ensemble machine learning classifiers are presented. The values were calculated by training and testing the ensemble classifier of logistic regression, random forest and gradient boosting.
(PDF)

S5 File Hyperparameter values for machine learning models. The hyperparameter values of machine learning model comparison study that were searched by grid-search method are presented.
(PDF)
