# Supplementary material for: Using machine learning for the personalised prediction of revision endoscopic sinus surgery
Source: PLoS One. 2022 Apr 29;17(4):e0267146. doi: 10.1371/journal.pone.0267146 (PMC9053825; doi:10.1371/journal.pone.0267146)
Supplement: S1 File — List of procedure and ICD10 codes that were used for identifying ESS patients and chronic diseases. (PDF) [file pone.0267146.s001.pdf]

File 1: Procedure and ICD10-codes.

Table A: Procedure codes that were used for identifying ESS patients. ESS = Endoscopic sinus surgery, CPT = Current procedural terminology.

CPT Code	CPT Code Description
DHB20	Polypectomy of internal nose
DMA00	Biopsy of maxillary antrum
DMB00	Endonasal trephine of maxillary antrum
DMB10	Radical maxillary antrotomy
DMB20	Functional endoscopic opening of maxillary antrum
DMB30	Transmaxillary excision of lesion of maxillary antrum
DMB35	Endoscopic extirpation of tumor or other change from maxillary sinus
DMC00	Removal of foreign body from maxillary antrum
DMW00	Intubation of maxillary antrum
DMW99	Other operation on maxillary antrum
DNA00	Biopsy of ethmoidal sinus
DNB20	Endoscopic ethmoidectomy
DNB30	Excision of lesion of ethmoidal sinus
DNB40	Sublabial rhinotomy
DNW99	Other operation on ethmoidal sinus and bone
DPA00	Biopsy of frontal sinus
DPA20	Trephination of frontal sinus
DPA25	Trephination of frontal sinus through nose
DPA35	Endoscopic extirpation of change or tumor from frontal sinus
DPB00	Partial excision of frontal sinus
DPB20	Obliteration of frontal sinus
DPW00	Intubation of frontal sinus
DPA10	Biopsy of sphenoidal sinus
DPA30	Sphenotomy
DPA40	Extirpation of change or tumor in the sphenoid sinus
DPC00	Removal of foreign body from frontal or sphenoidal sinus
DPC30	Extirpation of extensive change or tumor located in the region of multiple sinuses through open surgery
DPC35	Endoscopic extirpation of extensive change or tumor located in the region of multiple sinuses
DPW99	Other operation on frontal or sphenoidal sinus

Table B: ICD-10 codes that were used for identifying chronic diseases. ICD = International classification of diseases.

Disease	ICD-10 codes
Allergy	J30, J45.0
Asthma	J45
Cancer	C0-C97
Cardiovascular diseases	I00-I99
Chronic respiratory diseases	J40-J47, J80-J84, J90-94, G47.3
Diabetes	E10-E14.8
Gastroesophageal reflux	K21, R12, K44, K22.7
Immunodeficiency	B20, D80-D84
Mental disorders	F00-F99
Memory disorders	F00-F02, G30-, F11-F19
Mouth breathing	R06.5
Musculoskeletal diseases	M00-M99
Obesity	E65-E67
Obstructive sleep apnea	G47.3
