# Supplementary material for: Using machine learning for the personalised prediction of revision endoscopic sinus surgery
Source: PLoS One. 2022 Apr 29;17(4):e0267146. doi: 10.1371/journal.pone.0267146 (PMC9053825; doi:10.1371/journal.pone.0267146)
Supplement: S2 File — Keyword-based information extraction method was used for processing variables from free clinical texts. (PDF) [file pone.0267146.s002.pdf]

File 2: Key words mining from clinical texts.

Sets of disease specific key words (in finnish) were defined for the mining of patient variables from the clinical text (Table A). For example, the key words of diabetes were: 'diabetes', 'sugar', 'blood sugar' and 'insulin'. When a key word was found from clinical text, rule based inference identified cases that were related to negation, family history or good medical status.

Figures 1 and 2 present example steps of key word search for the cases of diabetes and NERD (Non-steroidal anti-inflammatory drug –exacerbated respiratory disease). First, input text is lower cased and tokenized. Key words are presented as stemmed form. Next, tokens from clinical text are compared to the set of key words. Let's assume that the number of characters of stemmed keyword is n. When n first characters of clinical text token and stemmed key word match (green square in Figures 1 and 2), the tokens before and after the matched key word are inputed for the rule based inference.

Two principles are applied for selecting tokens to the rule based inference. First, selected tokens should belong in same sentence as matched key word. Secondly, the distance from matched key word should be smaller than 10 tokens. Sentences are separated by finding period characters (blue square in Figures 1 and 2) from clinical text. The distance principle is applied only for cases where a key word sentence is long or periods are missing or unclear.

The rule based inference uses disease specific dictionaries for evaluating when matched key words relate to the negation of disease, family history or good medical status (Column "Rule based dictionary" in Table A). For example, Figures 1 and 2 present an example where rule based inference found the token 'no' before the key word (red square) meaning negation of disease.

Table A: Key words for the mining of patient variables from the clinical text and disease specific dictionaries for evaluating when matched key words relate to the negation of disease, family history or good medical status

Disease	Key word	Rule based dictionary	Extra rules
Allergy	cat	no, negative, mother, sister	
	birch	no, negative, normal	
	prick	no, negative, normal	
	skin prick test	no, negative, normal	
	rast	no, negative	Only "rast" or "rast-xxx"
Cancer	malignant	no	
	carcinoma	no	
	basalioma	no	
	cancer	mother, father, family	
	tumor	no, benign	
	melanoma		
Cardiovascular disease	hypertension	no, normal, mother, father, family	negation if [not known] in same sentence
	Coronary artery disease	no, normal, mother, father, family	negation if [not known] in same sentence
	coronary heart disease	no, normal, mother, father, family	negation if [not known] in same sentence
	aneurysm	no, normal, mother, father, family	negation if [not known] in same sentence
	cerebral hemorrhage	no, normal, mother, father, family	negation if [not known] in same sentence
	heart attack	no, normal, mother, father, family	negation if [not known] in same sentence
	angina	ei_	
	heart	normal, ok, ordinal, regular, good, balance, family, mother, father, no, clean, impeccable, clean, normal, health, calm, vibrant, symmetric, parents ordinal, okay, normal, compensation	
	rhythm	regular, steady, calm, silent, no, family	
Chronic respiratory disease	bronchitis	no, mother, father, family	
	copd	no, mother, father, family	
Diabetes	diabetes	family, father, mother	
	insuline		
	sugar	normal, ok, father, mother, father, candied, sugary sugar free, food, fat, diet	
	blood sugar	good, normal, check	
Memory disorder	memory problem	no	
	memory impairment	no	
	memory disorder	caregiver, wife, husband, mother	
	Alzheimer	caregiver, wife, husband, mother	
	dementia	work, working	
Mental disorder	depression		
	mood disorder		
	adhd		
	psychiatrist	works, job, working	
	behavioral		Same sentence should include: "disorder", "problem", "difficulties", "regulation", "agressive", "control" or "self destructive"
Musculoskeletal diseases	osteoporosis	no, mother, father, family	
	joint	no, normal, family, mother, father, calm, good, working, over-moving	
	tules		Only "tules" or "tules-xxx"
	back	no, mother, father, family, close relative	Should be: back pain, back ache, back problem, spinal cord injury, ankylosing spondylitis
Obesity	obesity	no, mother, father, family	
	fat	no, mother, father, family	
NERD	aerd	no	
	samter	no	
	aspirin	no	
	asa	no, mg	
Immunodeficiency or its suspicion	infectious disease doctor	no	
	susceptibility to infection	no	
	immune deficiency	no	

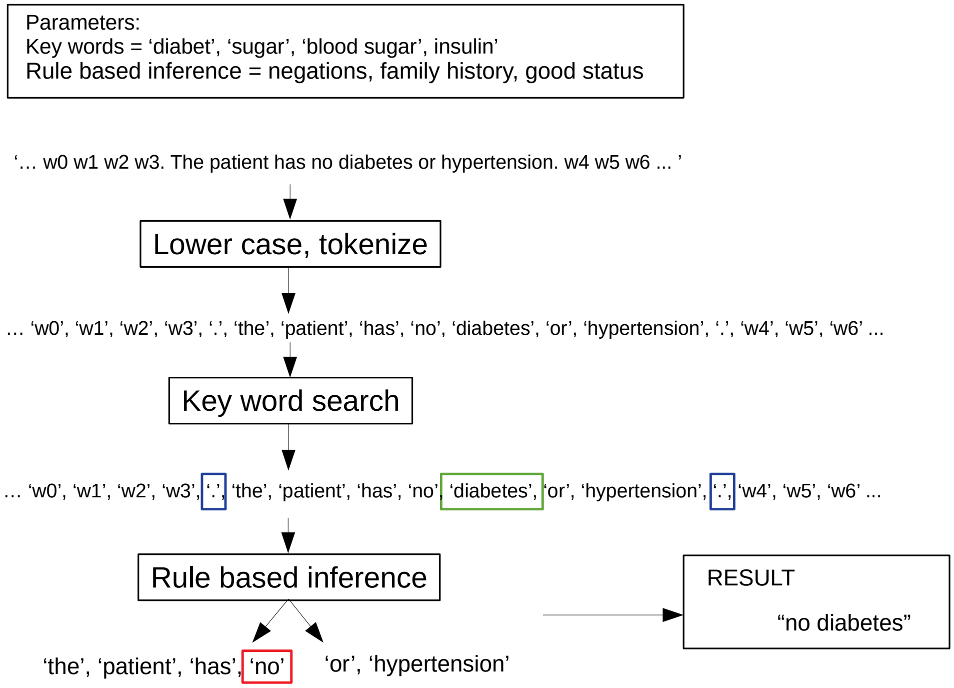

Figure 1: Example of keyword search process of diabetes disease from patient text

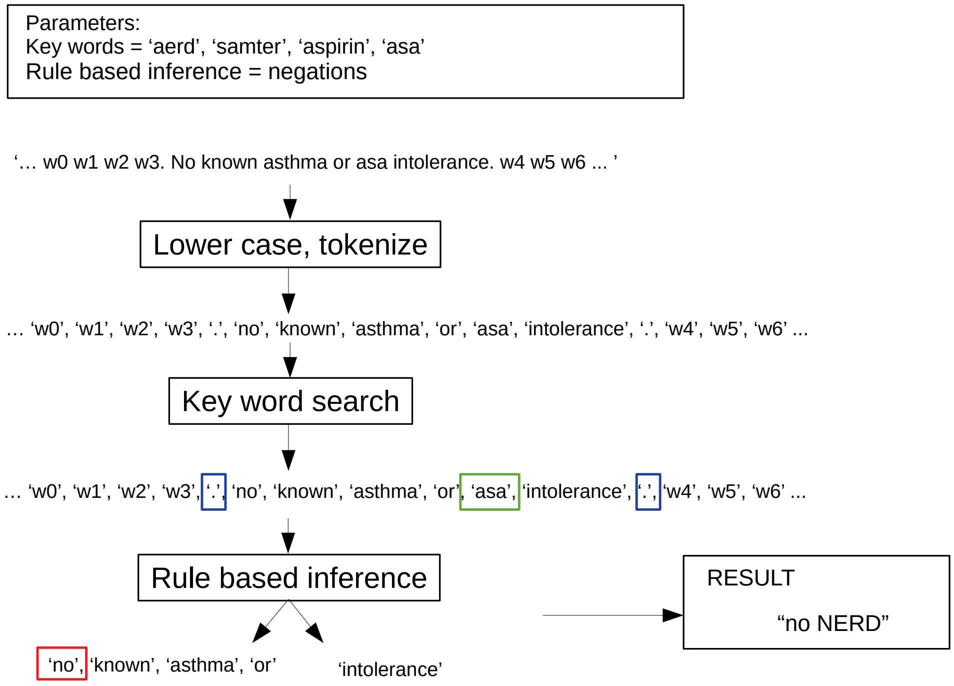

Figure 2: Example of keyword search process of NERD disease from patient text. NERD = Non-steroidal anti-inflammatory drug –exacerbated respiratory disease.
