# Supplementary material for: Using machine learning for the personalised prediction of revision endoscopic sinus surgery
Source: PLoS One. 2022 Apr 29;17(4):e0267146. doi: 10.1371/journal.pone.0267146 (PMC9053825; doi:10.1371/journal.pone.0267146)
Supplement: S3 File — Baseline performance values for machine learning classifiers are presented. The values were calculated by training and testing the classifiers when the labels of data were randomized. (PDF) [file pone.0267146.s003.pdf]

# File 3: Baseline machine learning classifier performance

Table A: AUROC and AUPRC values as a function of the number of variables for predicting revision ESS when models were trained with random labels (compare with Table 2). The results present baseline values for the models of the study. The variables were selected by sequential forward selection (SFS) method. Three models were used and the classifiers in these models were logistic regression (LR), gradient boosting (GB) and random forest (RF) for predicting revision ESS. AUROC = Area under the receiver operating characteristics curve, AUPRC = Area under the precision recall curve, ESS = Endoscopic sinus surgery.

| Number of variables | AUROC LR | AUROC GB | AUROC RF | AUPRC LR | AUPRC GB | AUPRC RF | F1 score LR | F1 score GB | F1 score RF |
|---|---|---|---|---|---|---|---|---|---|
| 1 | 0.501 | 0.494 | 0.472 | 0.146 | 0.154 | 0.155 | 0.214 | 0.049 | 0.211 |
| 2 | 0.511 | 0.502 | 0.480 | 0.177 | 0.157 | 0.166 | 0.219 | 0.069 | 0.197 |
| 3 | 0.513 | 0.479 | 0.475 | 0.151 | 0.157 | 0.156 | 0.232 | 0.087 | 0.210 |
| 4 | 0.507 | 0.483 | 0.480 | 0.149 | 0.153 | 0.158 | 0.227 | 0.082 | 0.204 |
| 5 | 0.503 | 0.490 | 0.476 | 0.147 | 0.160 | 0.148 | 0.216 | 0.078 | 0.198 |
| 6 | 0.499 | 0.483 | 0.478 | 0.149 | 0.151 | 0.148 | 0.222 | 0.105 | 0.179 |
| 7 | 0.501 | 0.485 | 0.481 | 0.146 | 0.148 | 0.152 | 0.224 | 0.085 | 0.186 |
| 8 | 0.498 | 0.472 | 0.486 | 0.143 | 0.142 | 0.149 | 0.213 | 0.097 | 0.189 |
| 9 | 0.491 | 0.484 | 0.467 | 0.142 | 0.151 | 0.145 | 0.204 | 0.075 | 0.188 |
| 10 | 0.497 | 0.483 | 0.475 | 0.145 | 0.163 | 0.156 | 0.204 | 0.071 | 0.193 |
| 11 | 0.502 | 0.488 | 0.489 | 0.147 | 0.159 | 0.149 | 0.206 | 0.070 | 0.193 |
| 12 | 0.506 | 0.482 | 0.477 | 0.146 | 0.155 | 0.151 | 0.220 | 0.061 | 0.180 |
| 13 | 0.506 | 0.487 | 0.470 | 0.146 | 0.153 | 0.147 | 0.217 | 0.099 | 0.178 |
| 14 | 0.514 | 0.478 | 0.476 | 0.152 | 0.170 | 0.144 | 0.224 | 0.086 | 0.185 |
| 15 | 0.514 | 0.471 | 0.483 | 0.152 | 0.140 | 0.144 | 0.229 | 0.047 | 0.190 |

Table B: AUROC and AUPRC values as a function of the number of variables for predicting revision ESS when models were trained by using the true labels (compare with Table 1). The variables were selected by Sequential Forward Selection (SFS) method. Three models were used and the classifiers in these models were logistic regression (LR), gradient boosting (GB) and random forest (RF) for predicting revision ESS. AUROC = Area under the receiver operating characteristics curve, AUPR = Area under the precision recall curve, ESS = Endoscopic sinus surgery.

| Number of variables | AUROC LR | AUROC GB | AUROC RF | AUPRC LR | AUPRC GB | AUPRC RF | F1 score LR | F1 score GB | F1 score RF |
|---|---|---|---|---|---|---|---|---|---|
| 1 | 0.652 | 0.628 | 0.606 | 0.327 | 0.322 | 0.317 | 0.339 | 0.335 | 0.305 |
| 2 | 0.726 | 0.703 | 0.683 | 0.332 | 0.345 | 0.326 | 0.359 | 0.366 | 0.322 |
| 3 | 0.738 | 0.722 | 0.656 | 0.341 | 0.341 | 0.298 | 0.388 | 0.401 | 0.313 |
| 4 | 0.740 | 0.723 | 0.678 | 0.350 | 0.356 | 0.312 | 0.405 | 0.406 | 0.351 |
| 5 | 0.735 | 0.727 | 0.701 | 0.347 | 0.368 | 0.318 | 0.407 | 0.409 | 0.341 |
| 6 | 0.744 | 0.731 | 0.719 | 0.354 | 0.360 | 0.319 | 0.398 | 0.402 | 0.369 |
| 7 | 0.739 | 0.730 | 0.722 | 0.347 | 0.352 | 0.339 | 0.401 | 0.378 | 0.365 |
| 8 | 0.730 | 0.741 | 0.723 | 0.344 | 0.378 | 0.334 | 0.400 | 0.413 | 0.359 |
| 9 | 0.731 | 0.724 | 0.732 | 0.343 | 0.351 | 0.352 | 0.398 | 0.388 | 0.364 |
| 10 | 0.730 | 0.731 | 0.727 | 0.343 | 0.365 | 0.339 | 0.398 | 0.401 | 0.365 |
| 11 | 0.733 | 0.726 | 0.737 | 0.345 | 0.351 | 0.348 | 0.401 | 0.391 | 0.385 |
| 12 | 0.734 | 0.724 | 0.733 | 0.338 | 0.360 | 0.349 | 0.383 | 0.409 | 0.376 |
| 13 | 0.727 | 0.737 | 0.720 | 0.331 | 0.366 | 0.347 | 0.380 | 0.380 | 0.354 |
| 14 | 0.726 | 0.722 | 0.729 | 0.334 | 0.349 | 0.342 | 0.379 | 0.387 | 0.372 |
| 15 | 0.730 | 0.725 | 0.736 | 0.336 | 0.343 | 0.337 | 0.383 | 0.373 | 0.370 |

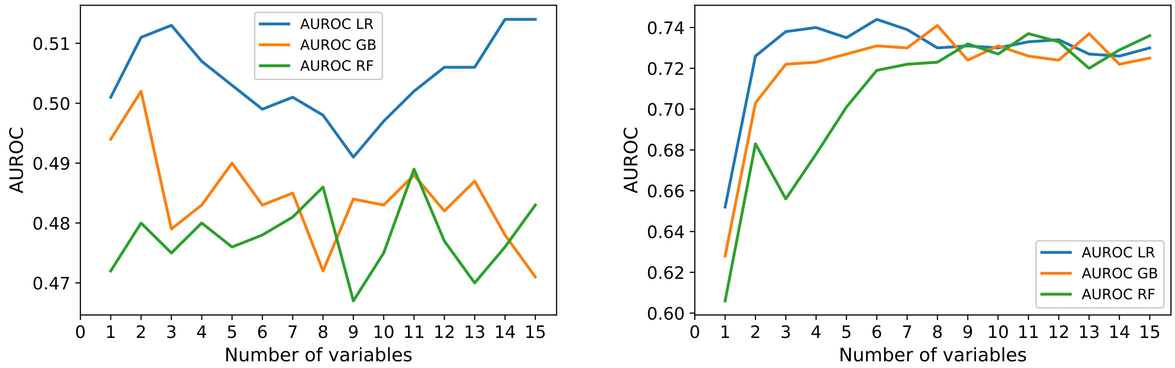


(a) (b)

Figure 1: **AUROC values as a function of the number of variables for predicting revision ESS.** Three models were used and the classifiers in these models were logistic regression, gradient boosting and random forest for predicting revision ESS. (a) performance when models were trained by using randomized label data (compare with Table 1), (b) performance when models were trained by using the true labels (compare with Table 2). AUROC = Area under the receiver operating characteristics curve, ESS = Endoscopic sinus surgery.
