# Supplementary material for: Using machine learning for the personalised prediction of revision endoscopic sinus surgery
Source: PLoS One. 2022 Apr 29;17(4):e0267146. doi: 10.1371/journal.pone.0267146 (PMC9053825; doi:10.1371/journal.pone.0267146)
Supplement: S4 File — Performance values for ensemble machine learning classifiers are presented. The values were calculated by training and testing the ensemble classifier of logistic regression, random forest and gradient boosting. (PDF) [file pone.0267146.s004.pdf]

File 4: Performance of ensemble model

Table A: Performance (AUROC, AUPRC, F1-score, sensitivity and specificity) values for ensemble classifier (ECLF) as a function of a number of variables when variables were selected by sequential forward selection (SFS). ECLF used soft voting method by aggregating logistic regression (LR), gradient boosting (GB) and random forest (RF) algorithms for predicting revision ESS. The hyperparameters of LR, RF and GB models were selected by grid-search method. The hyperparameters for grid-search were as follows: LR: C [.01, .1, 10, 100], penalty [l1, l2, elasticnet]; RF: max depth [2, 3, 4, 5, 6], number of estimators [4, 8, 32, 64]; GB: max depth [2, 3, 4, 5, 6], number of estimators [2, 4, 6, 8, 10, 12]. AUROC = Area under the receiver operating characteristics curve, AUPRC = Area under the precision recall curve, ESS = Endoscopic sinus surgery.

Number of variables	AUROC ECLF	AUPRC ECLF	F1 score ECLF	Sensitivity ECLF	Specificity ECLF
1	0.628	0.329	0.316	0.404	0.808
2	0.690	0.274	0.338	0.283	0.934
3	0.731	0.318	0.395	0.475	0.845
4	0.725	0.293	0.362	0.384	0.877
5	0.722	0.296	0.374	0.404	0.874
6	0.739	0.302	0.361	0.374	0.884
7	0.734	0.306	0.351	0.394	0.859
8	0.740	0.307	0.335	0.333	0.891
9	0.732	0.302	0.358	0.394	0.865
10	0.723	0.292	0.340	0.333	0.896
11	0.720	0.291	0.303	0.283	0.902
12	0.721	0.297	0.354	0.343	0.901
13	0.723	0.305	0.347	0.333	0.902
14	0.735	0.308	0.340	0.343	0.887
15	0.730	0.307	0.325	0.323	0.889
