# Supplementary material for: Using machine learning for the personalised prediction of revision endoscopic sinus surgery
Source: PLoS One. 2022 Apr 29;17(4):e0267146. doi: 10.1371/journal.pone.0267146 (PMC9053825; doi:10.1371/journal.pone.0267146)
Supplement: S5 File — The hyperparameter values of machine learning model comparison study that were searched by grid-search method are presented. (PDF) [file pone.0267146.s005.pdf]

File 5: Hyperparameter values for machine learning models

Table A: Selected hyperparameters for logistic regression model. The model was trained ten times with 1-15 variables.

Number of variables	C=0.01	C=0.1	C=1	C=10	C=100	Penalty=l1	Penalty=l2	Penalty=elasticnet
1	10	0	0	0	0	0	10	0
2	1	0	0	6	3	0	10	0
3	1	0	1	4	4	0	10	0
4	1	0	2	5	2	0	10	0
5	1	0	2	1	6	0	10	0
6	0	0	4	2	4	0	10	0
7	0	0	1	5	4	0	10	0
8	0	0	1	5	4	0	10	0
9	0	0	1	3	6	0	10	0
10	0	0	1	7	2	0	10	0
11	0	0	0	6	4	0	10	0
12	0	0	0	5	5	0	10	0
13	0	0	0	4	6	0	10	0
14	0	0	0	5	5	0	10	0
15	0	0	0	5	5	0	10	0

Table B: Selected hyperparameters for random forest model. The model was trained ten times with 1-15 variables.

Number of variables	bootstrap=true	bootstrap=false	max_depth=2	max_depth=4	max_depth=6
1	9	1	5	2	3
2	4	6	6	1	3
3	4	6	0	2	8
4	4	6	1	5	4
5	5	5	3	6	1
6	4	6	1	7	2
7	6	4	0	8	2
8	6	4	1	8	1
9	8	2	0	10	0
10	6	4	1	8	1
11	7	3	0	7	3
12	5	5	3	6	1
13	7	3	1	9	0
14	4	6	1	6	3
15	5	5	1	7	2

Table C: Selected hyperparameters for random forest model. The model was trained ten times with 1-15 variables.

Number of variables	max_features=auto	max_features=sqrt	min_samples_leaf=1	min_samples_leaf=2	min_samples_leaf=4
1	10	0	4	2	4
2	10	0	2	2	6
3	10	0	3	4	3
4	10	0	3	4	3
5	10	0	4	2	4
6	10	0	3	5	2
7	10	0	2	6	2
8	10	0	0	5	5
9	10	0	3	4	3
10	10	0	3	4	3
11	10	0	2	4	4
12	10	0	3	6	1
13	10	0	2	4	4
14	10	0	2	3	5
15	10	0	1	5	4

Table D: Selected hyperparameters for random forest model. The model was trained ten times with 1-15 variables.

Number of variables	min_samples_split=2	min_samples_split=5	min_samples_split=10	n_estimators=8	n_estimators=32	n_estimators=64
1	7	1	2	4	3	3
2	8	0	2	5	2	3
3	5	1	4	6	4	0
4	4	1	5	4	1	5
5	4	2	4	3	3	4
6	4	0	6	1	4	5
7	3	1	6	1	3	6
8	5	2	3	1	3	6
9	3	3	4	2	5	3
10	5	0	5	3	5	2
11	3	1	6	1	6	3
12	4	2	4	2	2	6
13	5	2	3	2	3	5
14	2	3	5	3	3	4
15	2	1	7	0	4	6

Table E: Selected hyperparameters for gradient boosting model. The model was trained ten times with 1-15 variables.

Number of variables	max_depth=2	max_depth=3	max_depth=4	max_depth=5	max_depth=6
1	2	2	5	1	0
2	1	6	2	0	1
3	0	7	2	0	1
4	0	2	5	3	0
5	0	2	4	2	2
6	0	3	3	3	1
7	0	3	4	1	2
8	0	3	5	1	1
9	0	0	5	3	2
10	0	1	7	0	2
11	0	2	6	0	2
12	1	1	4	3	1
13	0	1	6	2	1
14	0	1	7	2	0
15	0	3	3	4	0

Table F: Selected hyperparameters for gradient boosting model. The model was trained ten times with 1-15 variables.

Number of variables	n_estimators=2	n_estimators=4	n_estimators=6	n_estimators=8	n_estimators=10	n_estimators=12
1	3	1	0	1	0	5
2	1	0	1	4	1	3
3	2	0	1	0	1	6
4	2	0	1	4	2	1
5	1	0	1	3	1	4
6	1	1	2	1	2	3
7	1	1	2	0	0	6
8	1	2	0	1	3	3
9	3	1	4	1	1	0
10	2	2	1	1	2	2
11	1	1	2	4	0	2
12	2	2	2	2	0	2
13	2	2	1	3	1	1
14	2	1	1	2	2	2
15	1	2	5	0	0	2

Table G: Selected hyperparameters for gradient boosting model. The model was trained ten times with 1-15 variables.

Number of variables	learning_rate=0.005	learning_rate=0.01	learning_rate=0.02	min_child_weight=15	min_child_weight=16	min_child_weight=17
1	7	0	3	8	0	1
2	3	2	5	6	2	0
3	6	0	4	5	1	1
4	3	2	5	3	3	2
5	2	1	7	3	3	1
6	7	2	1	2	3	1
7	4	2	4	3	1	0
8	3	1	6	3	2	1
9	5	2	3	2	4	0
10	3	3	4	4	0	0
11	4	1	5	4	2	0
12	4	1	5	4	2	0
13	6	0	4	6	2	1
14	4	2	4	4	3	1
15	5	2	3	4	1	2

Table H: Selected hyperparameters for gradient boosting model. The model was trained ten times with 1-15 variables.

Number of variables	scale_pos_weight=2	scale_pos_weight=2.5	scale_pos_weight=3	subsample=0.7	subsample=0.8	subsample=0.9	subsample=1.0
1	1	3	6	4	1	2	3
2	2	3	5	7	1	1	1
3	4	3	3	1	6	2	1
4	3	3	4	4	3	2	1
5	4	2	4	3	3	3	1
6	1	2	7	5	2	2	1
7	5	2	3	4	3	2	1
8	2	3	5	2	5	2	1
9	4	1	5	4	4	1	1
10	5	0	5	5	3	1	1
11	5	3	2	3	2	4	1
12	3	1	6	5	2	2	1
13	5	2	3	2	4	3	1
14	3	4	3	5	1	2	2
15	3	2	5	1	6	1	2
